# Traversing Shifting Sands—the Challenges of Caring for Someone With Alzheimer's Disease and the Impact on Care Partners: Social Media Content Analysis

**DOI:** 10.2196/55468

**Published:** 2025-02-18

**Authors:** Kristian Steen Frederiksen, Julie Hahn-Pedersen, Rebecca Crawford, Ross Morrison, Rose Jeppesen, Lynda Doward, Wendy Weidner

**Affiliations:** 1 Department of Neurology Danish Dementia Research Centre Copenhagen University Hospital, Rigshospitalet Copenhagen Denmark; 2 Department of Clinical Medicine Faculty of Health and Medical Sciences University of Copenhagen Copenhagen Denmark; 3 Novo Nordisk A/S Soeborg Denmark; 4 RTI Health Solutions Manchester United Kingdom; 5 Alzheimer’s Disease International London United Kingdom

**Keywords:** Alzheimer disease, caregiver, burden, health-related quality of life, social media

## Abstract

**Background:**

Social media data provide a valuable opportunity to explore the effects that Alzheimer disease (AD) has on care partners, including the aspects of providing care that have the greatest impacts on their lives and well-being and their priorities for their loved ones’ treatment.

**Objective:**

The objective of this social media review was to gain insight into the impact of caring for someone with AD, focusing particularly on impacts on psychological and emotional well-being, social functioning, daily life and ability to work, health-related quality of life, social functioning, and relationships.

**Methods:**

We reviewed social media posts from 4 sources—YouTube (Google), Alzheimer’s Association, Alzheimer Society of Canada, and Dementia UK—to gain insights into the impact of AD on care partners. English-language posts uploaded between May 2011 and May 2021 that discussed the impact of AD on care partners were included and analyzed thematically.

**Results:**

Of the 279 posts identified, 55 posts, shared by 70 contributors (4 people living with AD and 66 care partners or family members), met the review criteria. The top 3 reported or observed impacts of AD discussed by contributors were psychological and emotional well-being (53/70, 76%), social life and relationships (37/70, 53%), and care partner overall health-related quality of life (27/70, 39%). An important theme that emerged was the emotional distress and sadness (24/70, 34%) associated with the care partners’ experience of “living bereavement” or “anticipatory grief.” Contributors also reported impacts on care partners’ daily life (9/70, 13%) and work and employment (8/70, 11%). Care partners’ emotional distress was also exacerbated by loved ones’ AD-related symptoms (eg, altered behavior and memory loss). Caregiving had long-term consequences for care partners, including diminished personal well-being, family and personal sacrifices, loss of employment, and unanticipated financial burdens.

**Conclusions:**

Insights from social media emphasized the psychological, emotional, professional, and financial impacts on individuals providing informal care for a person with AD and the need for improved care partner support. A comprehensive understanding of care partners’ experiences is needed to capture the true impact of AD.

## Introduction

Alzheimer disease (AD) is the most common form of dementia [1]. Worldwide, an estimated 55 million people were living with AD in 2019, and by 2050 a projected 139 million people will be living with AD [2]. Common symptoms of AD include memory impairment, disorientation, loss of function, and mood or behavior changes [3]. In the final and most severe stage of dementia, cognitive and functional abilities are significantly impaired, resulting in an increasing need for care and support [4]. Most individuals caring for a person living with AD are informal care partners (ie, a relative or a friend), and globally more than 70% of informal care hours are provided by women [5].

Caring for someone with AD can impact on care partners’ health-related quality of life (HRQOL) and emotional well-being [6-10]. Care partners experience an increased burden and more distress as AD severity increases [11-14] and may experience a greater burden than care partners for individuals with other chronic diseases [15]. There is also evidence that care partners experience greater activity impairment, absenteeism (ie, absence from work), impairment related to presenteeism (ie, reduced productivity while at work), and overall work impairment than individuals who are not care partners [16]. Effective disease-modifying treatments for AD are beginning to emerge, representing the potential to address the unmet needs for those living with AD and their care partners. To inform treatment goals in AD and fully characterize the potential value of emerging treatments, it is important to understand the practical, emotional, and financial impacts of the disease, particularly on loved ones providing informal care.

Previous research has evaluated the indirect cost implications of providing informal care to people living with AD or evaluated the caregiving burden using clinical outcome assessments [5,6,16,17]. However, these evaluations do not represent the lived experiences of care partners, the aspects of providing care that have the greatest impacts on their lives and well-being, or their priorities for their loved ones’ treatment. Social media data provide a valuable opportunity to explore the impacts that AD has on care partners outside the formal research context. The objective of this study was to gain insight into the impact of caring for someone with AD, with a particular focus on impacts to daily activities, psychological and emotional well-being, HRQOL, work and finances, social functioning, and relationships.

## Methods

We conducted an iterative pragmatic review of publicly available social posts shared on global platforms (eg, YouTube [Google]), as well as advocacy websites and independent blogs and forums of interest, with relevant data on the care partner experience of AD.

### Data Identification and Collection

The following parameters were used to guide the manual identification and collection of social media data:

1. Date range: A historical search of social media posts sourced from May 2011 to May 2021.

2. Language: English language only.

3. Media types: YouTube, news blogs, patient organizations, forums, and comments.

4. Different combinations of keywords were used to optimize the approach for identifying social media posts relevant to the project objectives. Key search terms and potential search strategies are presented in Table 1.

**Table 1:** Social media review key search terms and potential search strategies.

Concept	Key search terms	Example potential search strategy^ a^
Care partner experience	“caregiver”, “care-partner”, “carer”, “family*”, “husband”, “wife”, “story”, “journey”, “narrative”, “experience”, “diary”, “blog”, “forum”	(caregiver OR care-partner OR carer OR family OR husband OR wife) AND (story OR journey OR narrative OR experience OR diary OR blog OR forum)
AD^b^	“Alzheimer*”, “dementia”, “senile”, “mild cognitive impairment”, “early onset dementia”, “cognitive dysfunction”, “neurocognitive disorder”, “cognitive impairment”, “memory clinic”	(Alzheimer* OR dementia OR senile OR mild cognitive impairment OR early onset dementia OR cognitive dysfunction OR neurocognitive disorder OR cognitive impairment)
Impact of AD on care partner	“impact”, “burden”, “effect”, “quality of life”, “family life”, “family activities”, “relationships”, “health”, “money”, “money worries”, “expense”, “expensive”, “cost”	(impact OR burden OR effect OR quality of life OR family life OR family activities OR relationships)

^a^Boolean operators were used where supported by search function.

^b^AD: Alzheimer disease.

In addition to YouTube, a total of 6 advocacy websites were identified as containing potentially relevant data; the terms and conditions of each website were reviewed. Only publicly accessible videos and comments were reviewed from YouTube; that is, no videos that required logging into YouTube or required the researcher to subscribe were collated. Each of the advocacy websites was contacted to ask for permission to include the data posted on their website in the review. In total, 3 advocacy websites granted permission, and 3 either refused permission or were unresponsive to the request. Only advocacy websites that granted permission to be included in the review—Alzheimer’s Association, Alzheimer Society of Canada, and Dementia UK—were accessed.

The target population for review included people with a self-reported diagnosis of AD and care partners or family members of people with a self-reported diagnosis of AD. Video footage and discussion blogs were manually reviewed by experienced qualitative researchers to determine eligibility for inclusion in the review. The prespecified eligibility criteria for the social media posts are presented in Textbox 1.

**Social media post inclusion criteria.**

- The contributor has a self-reported diagnosis of mild cognitive impairment or dementia due to Alzheimer disease (AD) or is a self-reported carer or care partner or a family member of someone with mild cognitive impairment or dementia due to AD.
- At the time of the post, the contributor was an adult (aged 18 years or older). Where age was not reported, the contributor’s adult status was researcher-determined based on post content and video images.
- The post is relevant to the contributor’s experience of caring for someone with AD, including the impact of their AD treatments or management.
- Posts are in the English language.
- Post was uploaded between May 2011 and May 2021.

### Data Extraction

The social media posts (ie, videos and comments posted on YouTube and patient stories posted on the 3 selected advocacy websites) were reviewed manually by experienced qualitative researchers, and relevant social media data were extracted and recorded within a Microsoft Excel spreadsheet for analysis. Each post, blog, video, or comment included in the review was given a unique source identification number, and each contributor was assigned a unique contributor identifier. An initial deductive approach used a top-level coding framework to organize the extracted social media data by key themes, such as symptoms, disease history, HRQOL impacts, and treatment experience. Throughout the data extraction process, the research team met regularly to review and reflect on the data extracted. These points of researcher reflection included cross-checking contributor characteristics to ensure that any contributors who had posted across multiple platforms had not been double counted and only new information had been extracted in the second record, as well as a topline quality review of the data extracted. These sessions also allowed for the researchers to harmonize on the data extraction process.

### Data Analysis

Once all the social media data had been extracted and categorized using the top-level framework, the data were reviewed qualitatively by 2 researchers (RC and RM). A reflexive thematic analysis [18] of the aggregated social media data extracted from the posts was conducted, in which a theme was described as content that captured data relevant to the research objectives. Specifically, a deductive approach was initially used to code the social media data to address the study objectives, and descriptive codes were used to capture the concepts of interest. The coded data were then inductively analyzed within and across each category, with new codes added as concepts emerged from the data. This “bottom-up” approach is synergetic with the unsolicited and unregulated nature of social media data, as the approach allows the data to direct the analysis and the identification of new and important findings not predetermined by the research scope. Concepts were then used to generate patterns in the way that care partners described their experiences and perceptions related to looking after someone with AD. Patterns that emerged within and across the data were used to develop themes, with care partner experiences and perceptions summarized within each key theme.

The analysis approach and findings were reviewed by a third independent researcher (LD), and the interpretation of the social media data and the identification of themes and concepts were discussed to ensure the confirmability of the results. Once the themes and concepts were agreed, where appropriate, descriptive summaries were accompanied by frequency counts (and percentages) of the number of contributors discussing a specific theme.

Demographic information (eg, gender and age) was extracted, along with any accompanying disease information, where available. Notably, because social media data exist outside the research context, key demographic and diagnostic characteristics were not always available for contributors.

### Ethical Considerations

This study was submitted to RTI International’s institutional review board committees for ethical approval and was determined not to constitute research involving human participants (STUDY00022118). No informed consent was obtained, as the data analyzed in the study are publicly available. No personally identifiable information was included in the analyses. Modified quotations (supporting statements) are used to illustrate the key themes that emerged from the social media data. Modified quotations are used to ensure that the anonymity of the contributor is maintained, and their identity is protected. There were no participants to be compensated, as this study did not constitute research involving human participants.

## Results

### Post and Contributor Characteristics

**Figure 1:**
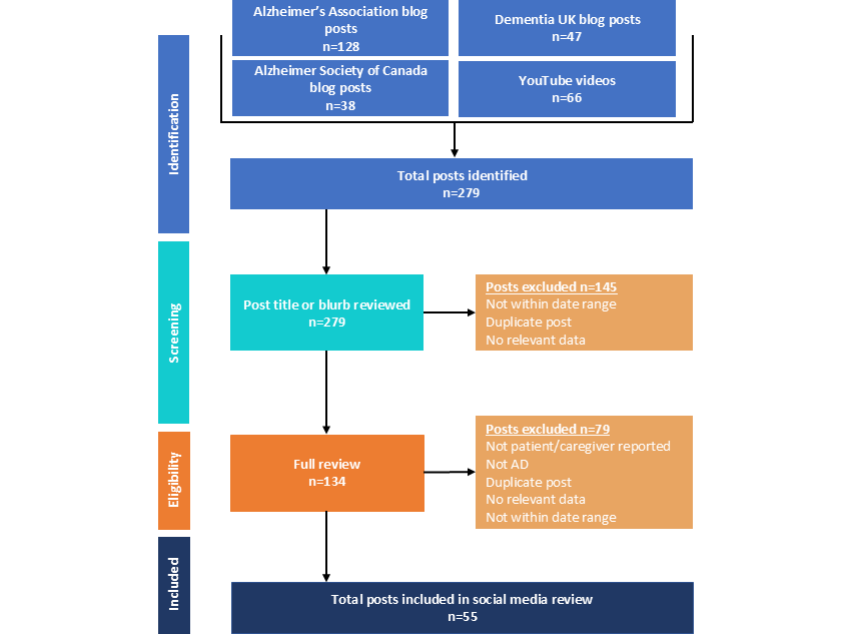
Social media post identification flow chart. AD: Alzheimer disease.

Of the 279 social media posts identified, 55 met the review criteria (21 blog posts, 21 videos, and 13 comments) (Figure 1); the remaining posts were excluded from the review because they did not meet the criteria for inclusion (eg, no relevant data included within the post, outside the prespecified date range). The 55 posts were shared by 70 contributors (4 people living with AD and 66 care partners or family members) who discussed self-reported or observed impacts of AD on care partners and family members. Table 2 provides the gender and age of contributors by contributor type (ie, care partner or family member and person living with AD) and for the total sample. Contributor age at the time of the social media post was not widely available; 90% (63/70) of contributors did not report their age in the social media post.

**Table 2:** Summary of social media contributor sample characteristics (the target population for review included care partners or family members of people with a self-reported diagnosis of Alzheimer disease; however, some contributors were individuals with Alzheimer disease who commented on care partners’ experiences. These posts were included in the review. Percentages are based on nonmissing data).

Characteristics			Contributor type				Total contributor sample (N=70)
			Person with AD^a^ (n=4^b^)		Care partner or family member (n=66^b^)		
**Self-reported gender, n (%)**							
	Women	2 (50)		40 (68)		42 (67)	
	Men	2 (50)		19 (32)		21 (33)	
	Missing	0^c^		7^c^		7^c^	
**Age at social media post, n (%)**							
	<65 years	1 (100)		4 (67)		5 (71)	
	≥65 years	0 (0)		2 (33)		2 (29)	
	Missing	3^c^		60^c^		63^c^	
	Age range (reported sample)	57		21-73		21-73	

^a^AD: Alzheimer disease.

^b^The contributor who self-identified as both a person living with Alzheimer disease and a care partner is included in the care partner or family member contributor cohort.

^c^Percentages are based on nonmissing data.

### Impact of AD on Care Partners

**Figure 2:**
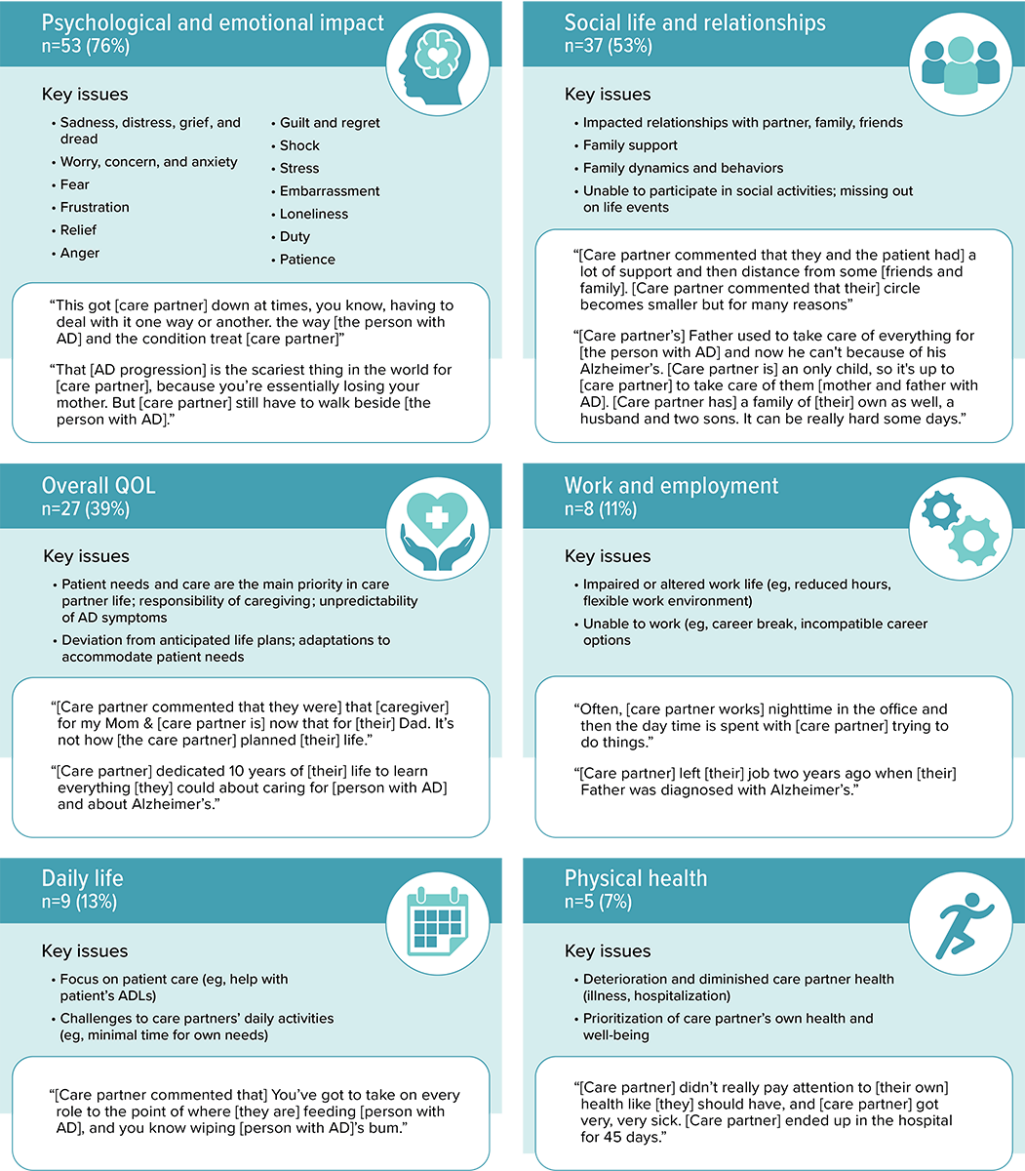
Key impacts of Alzheimer disease on care partners and family members reported by a person living with Alzheimer disease, care partner, or family member (n=70). AD: Alzheimer disease; ADL: activity of daily living; QOL: quality of life.

A range of impacts of AD on care partners and family members was discussed in the social media posts shared by the 70 contributors. The 3 most frequently reported or observed impacts of AD on care partners and family members were noted in relation to care partners’ psychological and emotional well-being (53/70, 76%), their social life and relationships (37/70, 53%), and their overall HRQOL (27/70, 39%). Contributors also reported impacts on care partners’ daily life (9/70, 13%), work and employment (8/70, 11%), and physical health (5/70, 7%). The key impacts of caring for someone with AD on care partners and family members that emerged from the social media data are summarized in Figure 2.

#### Emotional and Social Impacts

Care partner or family member contributors discussed a range of emotions experienced in relation to caring for someone with AD. Contributors noted feelings of frustration, concern, and fear in relation to the progressive symptoms of AD (eg, memory loss and delusions) and subsequent changes in behavior (eg, repetitive questions and aggression). The behavioral symptoms of people living with AD had a substantial impact on care partners and family members; 6 out of 70 (9%) care partner or family member contributors reported that the person living with AD had become aggressive and displayed violent behavior toward them. Furthermore, 1 contributor noted the need for patience when caring for someone with AD, and another highlighted that the primary care partner experience was a lonely existence regardless of support from family and friends.

The most profound impact on the psychological and emotional well-being of care partners and ‌‌family members was the emotional distress and sadness (24/70, 34%) associated with the phenomenon of “living bereavement” or “anticipatory grief,” that is, grieving the loss of a person even before their physical body dies [19]. The deterioration of the memories and personhood for the person living with AD was a traumatic and heartbreaking experience for care partners and family members, who had to witness the gradual loss of their loved one. Several contributors noted that, while the person living with AD was still alive, they were grieving their loss due to the significant changes in the person’s mental health as well as their cognitive and physical functioning. This was particularly salient for care partners and family members of people with early-onset AD, who “lost” their loved one earlier than anticipated; one family member described feeling that his mother had been “missing” for most of his life. The final stages of AD were also distressing for care partners and family members, who described the person living with AD as a “shell” that was simply waiting to “shut down.” Their loved one’s death presented care partners and family members with contrasting emotions—relief that their duty of care was now complete but sadness because the last vestiges of the person were gone. One care partner noted that the grief they experienced once their loved one had died was different from the “living grief” they had endured while the person had been alive.

Over 50% of the contributors (37/70, 53%) noted that caring for someone with AD had an impact on the social life and relationships of the care partner or family member. The impact of AD on the relationship between the care partner and the person living with AD was discussed by 9 out of 70 (13%) care partners or family member contributors, specifically the strain on or deterioration of this relationship. For care partners of parents with AD, this entailed a loss of their parental relationship and its associated support system, whereas for care partners of spouses with AD, this meant the loss of intimacy with their partner. Another key theme was the impact of care responsibilities on the relationships between the care partner or family member and their wider family (9/70, 13%). The duty of care for the person living with AD took precedence in family life; contributors noted that they would have to “sacrifice” their own family needs to accommodate their care partner obligations. However, care partner or family member contributors stressed the importance of family support (8/70, 11%) for the care partner, particularly shared family responsibility for care for the person living with AD, which helped to strengthen familial bonds. Changes in family dynamics were another component that emerged from the social media data in relation to the impact of AD on the social life and relationships of care partners and family members (6/70, 9%). Care partners and family members experienced a role change within the family unit, and family members were expected to assume additional responsibilities to adjust to the new family “norm.” Care partners and family members discussed the impact that their care responsibilities had on their social life (2/70, 3%). One care partner commented on changes to their relationships with friends; although some friends provided support, others did not; ultimately, their social circle decreased in size.

#### Impact on Daily Life and Ability to Work

Out of 70, 9 (13%) contributors commented on the detrimental impacts on care partners’ and family members’ daily lives because of the responsibility of caring for a person living with AD. Direct responsibility for the self-care needs of the person living with AD (eg, feeding, bathing, and toileting) was noted by 5 (7%) contributors, whereas 1 (1%) contributor commented that they provided assistance to the person living with AD to enable the individual to perform their own daily activities. Contributors also reflected on the challenges experienced by care partners and family members to perform their own daily activities (2/70, 3%), including interference resulting from the person living with AD’s cognitive symptoms (eg, forgetting where they had moved objects) or the minimal time available for care partners and family members to focus on their own needs.

Contributors commented on the disruption that caring for a person living with AD had on their ability to work (8/70, 11%). Half of these contributors (n=4) noted that they had to stop working or were unable to work because of their care partner duties. For others (n=4), it necessitated a change to the structure of their work life, such as reduced working hours, working from home, less work-related travel, and flexible working hours to accommodate the care needs of the person living with AD. Although some employers were able to accommodate the need for a flexible work-life approach, this was not always possible. Care partners noted that there were limited career choices that would permit the flexibility needed to provide care.

#### Impact on Well-Being and HRQOL

The stress or burden of caring for a person living with AD was attributed to a deterioration in the care partners’ own physical health (3/70, 4%). The prioritization of the person living with AD’s well-being resulted in cases of care partner illness, hospitalizations, and diminished well-being (2/70, 3%). However, 2 care partners out of 70 (3%) recognized the importance of prioritizing their own health, as well as the health of the person living with AD, and made positive lifestyle changes (eg, increased exercise and reduced alcohol consumption) to maintain and improve their physical health. This positive approach was considered by 1 care partner out of 70 (1%) to be the most effective plan to provide long-term care for her husband.

Care partners’ and family member contributors’ overall HRQOL and anticipated future life plans were hindered by their responsibility as a care partner and the needs of the person living with AD. Care partner and family member contributors (9/70, 13%) noted that the responsibility of caring for a person living with AD was multifaceted as it required balancing their care responsibilities with their family roles and obligations, arranging additional support or developing a contingency plan if they were incapacitated for any reason, and handling the logistical duties associated with the person living with AD’s end of life (eg, reviewing wills, insurance policies, and mortgages). Caring for the needs of the person living with AD became the main priority in the care partner’s life (6/70, 9%) and, according to some contributors, required complete dedication “24 hours, 7 days a week.” Furthermore, 1 care partner contributor noted that she had dedicated 10 years of her life to self-education on AD and caring for her partner. However, the progressive nature of AD and the unpredictability of AD symptoms (6/70, 9%) meant that care partners had to continuously adapt to the changes in the behavior and symptoms of the person living with AD. The need to adapt to the person living with AD’s circumstance was highlighted by 4 contributors out of 70 (6%), who noted that they had made significant changes to their lives (eg, relocation and renovation of existing home) to accommodate the needs of the person living with AD. Ultimately, providing care for someone with AD had long-term consequences on the lives of care partners and family members; out of 70, 4 (6%) contributors discussed how their lives had deviated from their anticipated life path (eg, career change and moving back in with parent).

### Care Partners’ Experiences of AD Treatment and Management

Care partners and family member contributors discussed treatment hopes or expectations (3/70, 4%). The potential development of a cure for AD was appealing for contributors, as was the potential for a novel treatment that could slow disease progression. In addition, 2 care partner contributors commented on the importance of involving the person living with AD in treatment decisions. Contributors’ discussions pertaining to decision-making in AD management focused predominantly on the factors that premeditated the decision by care partners and family members as to whether the person living with AD required formal full-time medical care at inpatient care facilities (6/70, 9%). Key factors discussed in the difficult decision to transfer an individual with AD from informal “at-home care” to formal “inpatient care” included crisis events or the realization that the needs of the person living with AD and the disease progression could be accommodated only with inpatient facility care (4/70, 6%).

Among the 13 contributors out of 70 (19%) who commented on the burden on care partners and family members associated with AD treatments or management, three key themes emerged, such as (1) limited support for care partners (6/70, 9%), (2) financial challenges (5/70, 7%), and (3) travel burden (3/70, 4%). Contributors commented on the challenges associated with navigating different services and health care systems (eg, difficulties accessing treatments or management options and benefits); 3 contributors (4%) lamented the absence of a single point of contact for assistance with access to treatment or management information and support. For these contributors, the process of navigating the health care system was time-consuming and created additional stress for themselves and the wider family. This process was further complicated by the contributor-perceived limited support for the diverse population of people living with AD, such as non–English-speaking individuals, people with early-onset AD, and people with AD who live in rural areas. Difficulties experienced with navigating or accessing AD-specific care services were also a factor contributing to the substantial financial responsibility assumed by care partners and family members. Contributors noted that they had not anticipated or planned for the high costs associated with the care needs of a person living with AD (ie, full-time care, memory care facilities, and assisted living). Furthermore, 1 care partner highlighted the difficult choice between becoming a full-time carer for the person living with AD and having the ability to meet that person’s financial needs. Another aspect of the burden of AD treatment or management on care partners was the considerable time commitment required to accompany the person living with AD to their frequent medical appointments.

## Discussion

### Principal Findings

The social media data collected in this study yielded insights into the impact to care partners and family members of providing care for people living with AD and the management of the disease. While positive elements associated with providing care for a person living with AD were discussed in the social media data, the posts emphasized in particular the concept of “living bereavement” or “anticipatory grief” for care partners and with that the need for improved coping strategies and interventions to enable care partners to better manage this phenomenon. Anticipatory grief permeates the experience of the care partner, which can involve decades of providing care for the person living with AD [19]. Accordingly, previous research has highlighted that it is important to consider anticipatory grief in conceptual models of care partner burden to help clarify how different stressors influence individual care partner outcomes and to enhance the understanding of the care partner experience [20].

Care partners’ and family members’ candid posts also highlighted the psychological, social, and financial impairments associated with becoming a care partner. Contributors reflected on the role of caring for a person living with AD as a full-time commitment and responsibility in which the needs of the person living with AD were often prioritized to the detriment of the care partner’s own needs, family life, and well-being. Providing care could also result in potentially daunting financial challenges and concerns.

Care partners’ treatment hopes and expectations, as expressed in the social media posts, focused predominantly on the possibility of a curative treatment in the future but also on the immediate need for treatments that could slow or prevent disease progression. Care decisions focused principally on the difficult and often distressing determination by care partners or family members that the person living with AD was now at a stage where they would benefit from formal full-time medical care at an inpatient care facility. AD treatments and management strategies had a substantial burden on care partners and family members; contributors noted limited support available for care partners and the financial and travel challenges associated with the management and treatment needs of a person living with AD.

Findings from the social media review are broadly consistent with the published evidence on the experiences of care partners in AD. Notably, studies conducted in the United States, Brazil, China, and Japan have found that care partners of people living with AD experience depression, anxiety, and high levels of stress [7-10]. For example, a study evaluating the experiences of 125 US care partners and 60 demographically similar control participants (non–care partners) found care partners more likely than control participants to experience clinically significant depression (40% vs 5%), and 26% of care partners used antidepressants [7]. Our results provide additional and unique insights into the individual contributor experiences underlying the emotional impacts of providing informal care for a loved one with AD.

Of note, it has been estimated that time spent providing informal care for people with AD amounted to 82 billion hours in 2015, equivalent to more than 40 million full-time workers, and this number is projected to increase to the equivalent of 65 million full-time workers by 2030 [5]. Further, a majority of informal care partners in AD are women [5], who may experience disproportionate impacts to their professional lives and opportunities as they manage their care responsibilities. Insights from social media contributors complement this evidence by providing rich data on care partners’ lived experiences and the impacts they experience beyond time spent caregiving and indirect impacts to their work productivity.

Care partner contributors also expressed frustrations with navigating the health care system, in particular with the lack of a single point of contact for access to treatment. This consistent unmet need for people living with AD and their care partners is well-recognized in the AD community. Improving the AD care journey for individuals living with AD and their care partners was a strategic recommendation in the Alzheimer’s Disease International’s 2022 World Alzheimer’s Report, but implementation is still limited in many countries [2].

The emergence of novel therapies offers the promise of slowing or halting disease progression for people living with AD. To comprehensively evaluate the value of novel therapies, it is critical to first document the magnitude of the societal impact of AD; however, the qualitative experiences and perspectives of informal care partners in AD have been underrecognized and can be challenging to measure via traditional research methods. Insights from this social media review, which is underpinned by published evidence on the care partner experience, provide unsolicited insights into care partners’ experiences with the practical, emotional, and financial challenges they face, and provide directions for future research to explore these impacts through formal research channels. Ultimately, as the therapeutic landscape evolves, the perspectives of care partners, as well as people living with AD, should be integrated into value assessment frameworks in AD [21].

This social media review provides a unique approach to exploring the experiences and challenges associated with caring for people living with AD potentially not captured through traditional research methods. Nonetheless, limitations of the methodology are noted. First, social media data are not driven by any specific research question or objective; thus, they exist outside the formal research context. There is an inherent reliance on contributor self-identification and self-reported diagnoses that are not verifiable. Social media data were retrospectively collected from posts in the public domain; information pertaining to contributor demographic characteristics, location information and access to care, and the clinical characteristics of their loved one with AD or the level of insight about their disease was not consistently available. Limitations of sample size must be considered; some of the themes identified from the social posts are based on a small number of contributors, and the degree to which the theme is relevant to the wider populations of people living with AD and care partners could be challenged. Different platforms are used by different demographic groups, and there is a potential bias toward populations who are more likely to use the specific sites selected for the review, more likely to reside in the included countries, or more likely to be engaged and proficient with digital technologies. Furthermore, only English-language posts were reviewed. Finally, there is the potential for self-selection and publication bias.

### Conclusions

The candid social media posts from 70 contributors provide valuable insights on the responsibilities and complications of caring for someone with AD, as well as the shortcomings of current AD programs to sufficiently support care partners in successfully balancing the dichotomy of their caregiving role and their own personal needs. It is critical to understand the true impact on care partners of providing informal care—including difficulties with their health and well-being, emotional and social functioning, and professional lives—and the unmet needs that they face. With this understanding, we may begin to define AD care models to support care partners as they navigate the AD care journey with their loved one. Furthermore, characterizing the care partner experience positions the AD research community to define and measure therapeutic goals for novel treatments that not only allow people living with AD to maintain their cognitive functioning and daily activities but also enable care partners to balance their informal care responsibilities with their well-being and professional responsibilities.

## Abbreviations

AD: Alzheimer disease
HRQOL: health-related quality of life

## Data Availability

The data analyzed during this study are included in this published article.
